# Psychological distress and its associations with past events in pregnant women affected by armed conflict in Swat, Pakistan: a cross sectional study

**DOI:** 10.1186/s13031-015-0063-4

**Published:** 2015-12-10

**Authors:** Muhammad Naseem Khan, Anna Chiumento, Mukesh Dherani, Katie Bristow, Siham Sikander, Atif Rahman

**Affiliations:** Institute of Public Health and Social Sciences, Khyber Medical University, Phase 5 Hayatabad, Peshawar, Pakistan; Institute of Psychology, Health and Society, University of Liverpool, Liverpool, UK; Human Development Research Foundation, Islamabad, Pakistan

**Keywords:** Psychological distress, Pregnancy, Conflict, Traumatic events, Mental health, Social support, Daily life stressors, Maternal health, Pakistan

## Abstract

**Background:**

The public health significance of maternal mental health is well established. Armed conflicts expose populations to events that could have long-term negative consequences for mental health of pregnant women and their children. This study explores the prevalence and associated risk factors for psychological distress of women during pregnancy, including exposure to past conflict-related potentially traumatic events, in a population exposed to armed conflict in the Swat region of Pakistan.

**Methods:**

A community-based cross-sectional survey of 349 pregnant women in two union councils in Swat was conducted. Psychological distress was measured using the Self-Reporting Questionnaire (SRQ). Conflict-related potentially traumatic events (PTEs) were measured through an adapted version of the Harvard Trauma Questionnaire. Information was also collected on major life events (Life Events Checklist), social support (Multidimensional Scale of Perceived Social Support), and demographic and socio-economic variables.

**Results:**

Prevalence of current psychological distress was 38.1 % (95 % CI: 33.1, 43.3). Psychological distress was significantly associated with three or more potentially traumatic events (PTEs) experienced during the conflict (OR = 2.62, 95 % CI: 1.22, 5.61); three or more major life events in the year following the conflict (OR = 3.25, 95 % CI: 1.82, 5.82) and inversely associated with family support (OR = 0.91, 95 % CI: 0.88, 0.95).

**Conclusion:**

This is one of the first community based cross sectional surveys in Swat valley, Pakistan to assess the prevalence of psychological distress during pregnancy in an area affected by conflict. Over a third of women show evidence of significant psychological distress. Exposure to potentially traumatic events remained independently associated with psychological distress 1 year after conflict ended, suggesting that conflict exposure may have long-term impacts upon maternal mental health. Combining this with findings relating to the cumulative impact of major life-events, and the protective factor of family support highlights the importance of developing culturally appropriate psychological interventions accessible to pregnant women rebuilding their lives following conflict.

**Electronic supplementary material:**

The online version of this article (doi:10.1186/s13031-015-0063-4) contains supplementary material, which is available to authorized users.

## Background

The public health significance of poor maternal mental health is well established, recognised as the second leading cause of the burden of disease in women worldwide with potential long-term adverse impact on physical and cognitive development of infants [1]. High prevalence of perinatal mental health problems have been documented in Pakistan (ibid) [1], linked to poor physical development of the child [2]. This finding has also been confirmed in other studies conducted in low and middle income countries (LMIC) [3].

Factors such as older age in women, school going age in children [4] and history of trauma [5]; lack of social support [6, 7]; low levels of education [5]; and single, separated or widowed marital status [8] are identified as risk factors for perinatal mental distress. Armed conflicts expose populations to a range of factors that could have a negative impact upon their mental health. In conflict settings additional factors such as proximity to traumatic events, forced displacement, and destruction of property [7] potentially increase the risk of psychological distress.

The Swat valley in Pakistan experienced armed conflict beginning in September 2007 and ending in the autumn of 2011. Conflict involved widespread insurgency activity and military intervention including in 2008–9 internally displacing the 2.5 million people of the valley to live in camps, with relatives, or in rented accommodation across the region for approximately 4 months [9]. In July 2009 the Government declared the intervention a success, and the internally displaced persons (IDPs) began to return home. However, continued military presence created social unease [10], with sporadic armed conflicts continuing until the autumn of 2011. This conflict had adverse impacts on regional economy including the destruction of infrastructure such as schools and power station [11], health services, and social structures due to continued instability and displacement upon family and community ties [6].

This study sought to assess psychological distress in pregnant women in Swat valley one-year after the active conflict. Secondary aims were to explore the associations of factors such as exposure to potentially traumatic events, the occurrence of major life-events, and current levels of perceived social support with psychological distress.

## Methods

### Settings and population

Swat District has an estimated population of over 1.7 million [12], with a male to female ratio of 106:100. 86 % of the population live in rural areas with an average household size of 8.8 persons. Male literacy measured as education of 10 or more years is 43 %, compared to female literacy of 14 % [13].

This survey was conducted in two union councils of Swat District, Odigram and Qambar, with a combined population of approximately 50,000 living in 15 villages. A Union Council (UC) is the smallest administrative unit in a district with an average population of approximately 25,000 people. The UCs were selected because of accessibility to the district headquarter, and a functioning primary health infrastructure. The study was conducted in the catchment area of two basic health units (BHUs) – primary healthcare facilities staffed by a primary care physician, a Lady Health Visitor, a vaccinator, a midwife and 15–20 community-based Lady Health Workers (LHWs). The LHWs are community health workers trained to provide maternal and child healthcare and education. Each LHW is responsible for a catchment area of approximately 1,000 people or 150 homes and visits 5–7 homes daily, maintaining an official record of pregnancies through monthly routine visits to their allocated households [14, 15].

A community-based cross sectional survey was conducted between 1st September and 31st October 2012. The study population included all pregnant women residing in the catchment area of the LHWs attached to the two BHUs in Qambar and Odigram UC’s. Eighty to Eighty-five percent of the households are covered by LHWs in these settings [15]. The survey population was drawn from registered lists of pregnant women maintained by LHWs, including any pregnancy newly registered during survey conduct. Areas not covered by LHWs were therefore not included.

All registered pregnant women were approached by trained researchers, accompanied by the LHW who had obtained prior family agreement for the research team to visit. LHWs are well-respected local health professionals, trusted by families they work with and therefore appropriate to make initial introductions. The research team verbally explained the purpose of the study in the local language and written informed consent to participate in the study was obtained. Following this, researchers visited all the consenting women with the LHWs to administer the survey instruments. The LHW introduced the research team to the family who then reconfirmed consent prior to conducting the assessment. As far as possible, all the assessments were conducted in private.

### Instruments and procedures

#### Self-reporting questionnaire (SRQ)

The SRQ-20, developed by the WHO for use in primary care settings, was used to measure current psychological distress. It contains 20 questions relating to self-reported psychological distress experienced over the previous month. This instrument has been validated in Pakistan [16, 17], including the perinatal population [18]. Psychological distress was defined as a score of 9 or above on the SRQ following the work of Rahman et al*.* with a similar population in Pakistan [18].

#### Potentially traumatic events (PTEs)

The level of trauma exposure by pregnant women was assessed using an adapted version of the Harvard Trauma Questionnaire, which has been translated and culturally adapted into Urdu [19]. The questionnaire inquires about exposure to potentially traumatic events. For each potentially traumatic event there are four response categories depending upon proximity to the event and exposure level: (1) experienced, (2) witnessed, (3) heard, and (4) none. This instrument captured conflict-related potentially traumatic events faced by the population during periods of militancy in the region (September 2007 until September 2011). This encompasses the period in 2009 during which Pakistani military operations were conducted against the insurgents, including population evacuation and displacement across the region, their return and a continued period of unrest until 2011.

#### Multidimensional scale of perceived social support (MSPSS)

The Multidimensional Scale of Perceived Social Support (MSPSS) is a self-rating tool of perceived social support consisting of 12 questions rated on a 7 point scale developed by Zimet et al. [20]. Questions are grouped into 3 categories of support: from family, from friends and from significant other. The 7 point scale ranges from 1 “very strongly disagree” to 7 “very strongly agree”. This tool has been translated into Urdu and validated in the Pakistani population, showing good construct validity and internal consistency [21].

#### Life events checklist (LEC)

A locally validated Life Events Questionnaire was administered. This questionnaire was developed by Rahman [22] and adapted from a locally validated version of Life Events and Difficulties Schedule [16]. The questionnaire uses a checklist of binary response variables commonly associated with depression. These include: significant other made redundant, financial difficulties, housing difficulties, relationship difficulties such as arguments, and serious marital problems [22]. Questions on this checklist correspond to the time period September 2011 until September 2012, therefore measured the impact of post-conflict life events.

#### Socio-demographic characteristics

Socio-demographic information was collected relating to maternal/obstetric factors, family factors, and socioeconomic factors. Maternal/obstetric factors included age; education level (formal years of schooling); duration of current pregnancy; history of miscarriages, stillbirths or infant deaths; and if primigravida (first pregnancy). Family variables measured husbands education (formal years of schooling); number and ages of children in the family; family structure (extended structure with parents, in-laws and children living together in one household, or nuclear structure with only the married couple and their children). Socioeconomic status was assessed by LHWs using a 5 point Likert scale from 1 (rich) to 5 (poor) [22]. Although subjective, this brief technique provides an indication of socioeconomic status, drawing upon intimate LHWs knowledge about the community [22]. Socioeconomic status also included husband’s employment status and if the husband was absent from home due to work. Women’s financial empowerment was assessed by asking if the women were given money for day-to-day activities, and if they had authority to utilise that money according to their wishes [22]. Women who answered affirmatively to both questions were categorised as financially empowered.

### Training in use of instruments

The study lead (MNK) provided training to the research team to ensure all instruments were administered in a standardised manner to minimise interviewer bias. During the three day training all instruments were piloted in a different but socio-demographically similar area to the study site, with issues in their administration discussed and resolved with the study lead at the end of each training day. Piloting of the instruments was essential to ensure the instruments were administered consistently. Training also covered research ethics, responding to participant distress and referral to primary healthcare.

### Sample size calculations

Sample size was based on two objectives: (1) estimation of prevalence of psychological distress in pregnant women, and (2) association of psychological distress with exposure to conflict related potentially traumatic events.

For the first objective, assuming a prevalence rate of psychological distress of 40 % [21], an alpha of 0.05 and a precision of 0.05 a sample size of 369 pregnant women would allow prevalence estimation using the following formula: n = z^2^ p(1-p)/d^2^ where z is the statistic of confidence level, p is prevalence, and d is the precision using openEpi version 2.3.1 [23]. Assuming a non-response rate of 10 % for refusals and exclusion, a sample size of 410 was estimated.

To study associations between psychological distress in pregnant women and exposure to potentially traumatic events, the sample size was estimated based on the following assumptions: an alpha 0.05, power of 80 %, and a prevalence of potentially traumatic events in the population at 40 %, ratio of distressed vs non-distressed of 1:1.5, and an odds ratio of 2 as a measure of clinical significance. Using openEpi version 2.3.1 [23] a sample size of 300 was estimated.

### Statistical analysis

The main outcome variable, psychological distress, was dichotomised into a binary variable using a cut off of score 9 on SRQ [16, 18] based on which prevalence was calculated. Levels of social support were collected using the MSPSS questionnaire. MSPSS scores were used as a continuous variable. All potentially traumatic events (PTEs) were combined and categorisation was conducted based on number of events. Since the whole population was evacuated during hostilities (part of PTE score), a score of 2 or less was used as the reference category. Similarly life events were categorised into 2 groups based on numbers of events with category 2 and below was considered as a reference category. Socio-demographic data was also merged into 3 categories: 1 and 2 on the Likert scale represented high income; 3 represented middle income, and 4 and 5 low income.

Uni-variate analysis of the association between psychological distress and categorical data was conducted using Chi square (Fisher’s two-sided exact test was used if numbers in the cells <5), while for continuous data Mann–Whitney test was used (non-normal distribution of data). Associations were considered significant at 5 %. Logistic regression analysis of the association between psychological distress and all other variables was carried out. All those variables with p-value <0.1 on uni-variate analysis were entered in the logistic regression model. Collinearity between explanatory variables was assessed. All the analyses were carried out in SPSS version 20.

### Ethics

The study received ethical approval from Health Services Academy, Islamabad, Pakistan, and The University of Liverpool, UK. Informed written consent was obtained from participants following explanation of the study aims and procedures. In case of illiterate women all research information was explained verbally in the local dialect with a thumb print accepted in lieu of a signature, following standard practice in the context. In conflict settings informed consent has been recognised by the study team as a challenge, with strategies to address this outlined in a separate paper [24]. The right to withdraw from the study at any time without providing a reason was reinforced to all participants during informed consent and throughout survey conduct. Data confidentiality was assured, with all data accessible by the study lead only (MNK). Cases of severe depression and other health concerns requiring immediate treatment were referred to BHUs where trained medical staff managed cases accordingly.

## Results

**Fig. 1:**
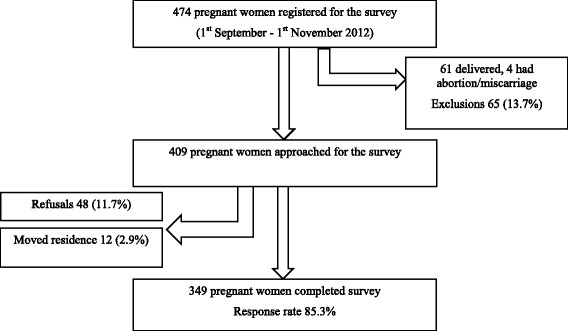
Flow diagram of survey sample

During the two-month period from 1^st^ September 2012 to 31st October 2012, 474 pregnant women were registered. Of these, 61 (12.9 %) delivered and 4 (0.8 %) had an abortion/miscarriage and were therefore excluded from the survey. Of the remaining 409 pregnant women 48 (11.7 %) refused to participate while 12 (2.9 %) migrated. Therefore, a total of 349 pregnant women were surveyed with a response rate of 85.3 % (Fig. 1). Of these women 49 % (172/349) were in their third trimester, 39 % (137/349) in their second trimester, and 10 % (40/349) in their first trimester.

### Prevalence of psychological distress

Applying a cut-off score of ≥9 to the SRQ to define psychological distress, the prevalence of psychological distress in pregnancy was 38.1 % (95 % CI: 33.1, 43.3), or 133/349 women surveyed.

### Demographic factors

Table 1 compares the demographic characteristics of the sample by psychological distress. On uni-variate analysis demographic factors significantly associated with psychological distress included previous pregnancy loss (*p* = 0.001) and primigravida (*p* < 0.0001), while infant death was marginally significant (*p* = 0.056). Having 2 or more children less than 7 years of age was significantly associated with psychological distress (*p* = 0.001), as was living in a nuclear family structure (*p* < 0.001). Not being financially empowered was also significantly associated with current psychological distress (*p* = 0.028).

**Table 1:** Socio-demographic characteristics and association with psychological distress

		Psychological distress	No psychological distress	*P*-value
		*N* = 133	*N* = 216	
		*n* (%)	*n* (%)	
Maternal/Obstetric factors				
Age	<= 19 years	18 (14)	50 (23)	0.073
	20–24 years	42 (32)	71 (33)	
	25–29 years	36 (27)	54 (25)	
	30+ years	37 (28)	41 (19)	
Education	No education	73 (55)	124 (57)	0.154
	1–5 years	30 (23)	32 (15)	
	6+ years	30 (23)	60 (28)	
Infant death	Any infant death	14 (11)	11 (5)	0.056
Pregnancy loss	Any pregnancy loss	49 (37)	44 (20)	0.001
Primigravida	First pregnancy	20 (15)	82 (38)	<0.001
Duration of pregnancy	1st trimester	15 (11)	25 (12)	0.994
	2nd trimester	52 (39)	85 (39)	
	3rd trimester	66 (50)	106 (49)	
Family factors				
Husband’s education	No education	40 (30)	75 (35)	0.510
	1–5 years	25 (19)	32 (15)	
	6+ years	68 (51)	109 (51)	
Family size	≥2 children younger than 7 years old	40 (30)	33 (15)	0.001
Family structure	Nuclear family	27 (20)	15 (7)	<0.001
Socioeconomic factors				
Husband’s employment	Regular employment	32 (24)	78 (36)	0.145
	Subsistence farming	6 (5)	12 (6)	
	Working on daily wages	64 (48)	83 (38)	
	Self employed	26 (20)	33 (15)	
	Unemployed	5 (4)	10 (5)	
Husband living away	≥6 months/year from home	16 (12)	34 (16)	0.337
Financial Empowerment	Not empowered	74 (56)	94 (44)	0.028
Socioeconomic status by LHW	High	20 (15)	45 (21)	0.348
	Middle	44 (33)	72 (33)	
	Low	69 (52)	99 (46	

### Potentially traumatic events

Traumatic experiences related to the conflict significantly associated with current psychological distress (Table 2) include: loss of shelter and logistics (*p* = 0.050); being close to death (*p* = 0.008); unnatural death of family member or friend (*p* = 0.005); stranger or strangers murder (*p* = 0.047) and torture (*p* = 0.005). Very few participants experienced the last two events. Witnessing imprisonment (*p* = 0.020) and hearing about sexual harassment (*p* = 0.027) were the only two events in ‘witnessed’ and ‘heard’ categories respectively that were associated with current psychological distress. The number of PTEs experienced was significantly associated with current psychological distress (*p* = 0.001) (Additional file 1: Table S1).

**Table 2:** PTEs significantly associated with psychological distress

Potentially Traumatic events	Psychological distress	No psychological distress	*P*-value
	*N* = 133	*N* = 216	
	*N* (%)	*N* (%)	
Experienced PTEs			
Loss of shelter and logistics	125 (94)	189 (88)	0.050
Being close to death	76 (57)	92 (43)	0.008
Unnatural death of family member or friend	27 (20)	21 (10)	0.005
Strangers’ murder	7 (5)	3 (1)	0.047^a^
Torture	12 (9)	5 (2)	0.005
Witnessed PTEs			
Imprisonment	36 (27)	36 (17)	0.020
Heard PTEs			
Sexual harassment	18 (14)	14 (7)	0.027
Number of PTEs experienced			
≤ 2	13 (10)	45 (21)	0.001
3	34 (26)	71 (33)	
4	38 (29)	59 (27)	
5	22 (17)	24 (11)	
6+	26 (20)	17 (8)	
Number of PTEs witnessed			
≤ 2	116 (87)	192 (89)	0.904^1^ ^a^
3	13 (10)	15 (7)	
4	3 (2)	6 (3)	
5	1 (1)	2 (1)	
6+	0 (0)	1 (1)	
Number of PTEs heard			
≤ 2	89 (67)	158 (73)	0.759
3	14 (11)	19 (9)	
4	9 (7)	11 (5)	
5	6 (5)	10 (5)	
6+	15 (11)	18 (8)	

*PTEs* Potentially Traumatic Events

^a^Fisher’s exact test where number in cells less than 5

### Life events

Table 3 shows that life events in the post-conflict years associated with current psychological distress included death or serious illness in a close relative (*p* = 0.027); livelihood problems (*p* = 0.037); financial problems (*p* = 0.001); troubled relations with close relatives/friends (*p* < 0.001); troubled marital relations (*p* < 0.001); worries about children’s health and education (*p* < 0.001); and family quarrels (*p* < 0.001). Three or more life events were highly significantly associated with current psychological distress (*p* < 0.001).

**Table 3:** Life’s events during the past one year and association with psychological distress

Major Life events during the past one year	Psychological distress	No psychological distress	*P*-value
	*N* = 133	*N* = 216	
	*n* (%)	*n* (%)	
You yourself or a close relative of yours had been ill or had an accident which led to hospitalization	70 (53)	101 (47)	0.287
Any of your close relatives died or committed suicide or had gotten seriously ill	64 (48)	78 (36)	0.027
Has anyone in your family had problems of livelihood	70 (53)	89 (41)	0.037
You or someone in your family has had any financial problem	87 (65)	101 (47)	0.001
You or someone in your family has had a change in social status	67 (50)	108 (50)	0.946
You yourself have had any problem with your residence	27 (20)	34 (16)	0.276
Your relations with any of your close relatives or friends have been troubled	41 (31)	28 (13)	<0.001
Your marital relations with your spouse have had problems	44 (33)	30 (14)	<0.001
You have been worried about your children’s problems	71 (54)	63 (29)	<0.001
You or other family members have had rows/quarrels amongst themselves	58 (44)	40 (19)	<0.001
Number of major life events in the post-conflict year			
≤2	24 (18)	98 (45)	<0.001
3-4	44 (33)	62 (29)	
5+	65 (49)	56 (26)

**Table 4:** Perceived social support and association with psychological distress

Perceived social support	Psychological distress	No psychological distress	*P*-value
	*N* = 133	*N* = 216	
	Mean (SD)	Mean (SD)	
There is a special person who is around when I am in need	4.89 (2.11)	5.93 (1.41)	<0.001
There is a special person with whom I can share my joys and sorrows	5.05 (2.11)	6.00 (1.37)	<0.001
I have a special person who is a real source of comfort to me	5.11 (2.11)	5.95 (1.44)	<0.001
There is a special person in my life who cares about my feelings	4.72 (2.27)	5.84 (1.58)	<0.001
Score on *Significant Other* subscale	19.77 ( 7.56)	23.72 ( 5.12)	<0.001
My family really tries to help me	4.80 (2.25)	6.01 (1.32)	<0.001
I get the emotional help and support I need from my family	4.92 (2.15)	6.06 (1.25)	<0.001
I can talk about my problems with my family	4.74 (2.28)	6.01 (1.38)	<0.001
My family is willing to help me make decisions	4.89 (2.19)	6.00 (1.39)	<0.001
Score on *Family* subscale	19.35 ( 8.22)	24.09 ( 4.78)	<0.001
My friends really try to help me	3.61 (2.10)	4.43 (1.89)	<0.001
I can count on my friend when things go wrong	3.20 (1.85)	4.04 (1.88)	<0.001
I have friends with whom I can share my joys and sorrows	3.58 (1.95)	4.25 (1.92)	0.002
I can talk about my problems with my friends	3.62 (1.96)	4.25 (1.93)	0.005
Score on *Friends* subscale	14.02 ( 6.92)	16.95 ( 7.04)	<0.001
MSPSS^a^ total score	53.14 (18.17)	64.76 (12.24)	<0.001

^a^MSPSS: Multidimensional scale of perceived social support

### Social support

Lower levels of perceived social support (Table 4) was significantly associated with current psychological distress (*p* < 0.001). Psychologically distressed women significantly perceived less social support on all three subscales of support from significant others (*p* < 0.001); family (*p* < 0.001) and; friends (*p* < 0.001).

### Logistic regression analysis

Logistic regression was conducted to examine the effects of demographic characteristics, PTE’s, major life events and perceived social support on current psychological distress adjusting for each other. All variables with *p* < 0.1 in uni-variate analysis were entered into the model through a priori criteria (Table 5).

**Table 5:** Multiple logistic regression analysis

Variables/factors		Odds ratio	95 % CI of Odds ratio	*P*-value
Maternal/Obstetric factors				
Age	<= 19 years			
	20 – 24 years	0.65	0.29, 1.46	0.294
	25–29 years	0.55	0.23, 1.34	0.189
	30+ years	0.53	0.20, 1.38	0.192
Infant death	Any infant death	1.75	0.65, 4.72	0.266
Pregnancy loss	Any pregnancy loss	1.06	0.58, 1.93	0.855
**Primigravida**	**First pregnancy**	**0.41**	**0.19, 0.89**	**0.024**
Family factors				
Family size	≥2 children younger than 7 years old	1.57	0.83, 2.96	0.166
**Family structure**	**Nuclear family**	**3.61**	**1.53, 8.52**	**0.003**
Socioeconomic factors				
Financial Empowerment	Empowered	0.98	0.58, 1.67	0.938
**3 or more PTEs experienced**		**2.62**	**1.22, 5.61**	**0.013**
**3 or more major life events in the post-conflict year**		**3.25**	**1.82, 5.82**	**<0.001**
Current social support				
**Score on** ***Family*** **subscale**		**0.91**	**0.88, 0.95**	**<0.001**
Score on *Friends* subscale		0.97	0.94, 1.01	0.128

(Significant factors are bold)

*PTEs* Potentially Traumatic Events; *CI Confidence Interval*

Psychological distress remained significantly associated with nuclear family structure, three or more major life events in the previous year; and experiencing three or more PTEs. Conversely, having higher levels of perceived social support from family members was significantly inversely associated.

## Discussion

This study confirms that psychological distress remains a major issue in pregnant women even one year after resolution of conflict in Swat Valley. The prevalence of current psychological distress in a community-based sample of pregnant women was 38.1 %. Current psychological distress was positively associated with exposure to three or more potentially traumatic events during the years of the conflict, spanning 2007 to 2011. There was a threshold effect with levels of psychological distress not manifesting until at least experiencing 3 or more potentially traumatic events, which have more than 2.5 time impact on psychological distress. Psychological distress was similarly independently associated with three or more major life events in the year after the conflict. Perceived family support and living in a joint family was protective.

The main strength of this study was by being a community-based study it reflects the true prevalence of psychological distress in the population. Centre-based studies may be misleading in post-conflict situations as many women do not have access to perinatal care [25]. We feel that the Lady Health Workers’ lists are comprehensive of their catchment areas, as they are from the local community and have first-hand information about all households under their care. However, the population in this survey live in close proximity to a major city, therefore might not be representative of women living in the more inaccessible rural areas. Another potential limitation is that the study could not systematically assess temporal relations between exposure and outcome due to the cross-sectional study design. Socioeconomic status and educational levels were not included in the final model of analysis as a priori criteria of including a variable in the model with *p* < 0.1 on uni-variate analysis was used. This could be a potential limitation. Additionally, recall bias may be present when asking about exposure to events that occurred 2–3 years prior. It can be argued, however, that given the magnitude of the events this was unlikely. A further limitation is the potential for negative recall bias where due to cognitive distortions associated with psychological distress women may be more likely to recall the exposure.

Pregnant women are identified as a social group at increased risk of psychological problems during humanitarian emergencies [6]. Given the importance of psychological health during pregnancy for maternal and child health outcomes [1, 2] it is important to understand the specific psychological health risk factors that affect pregnant women exposed to conflict. The higher prevalence of psychological distress during pregnancy found in this study concurs with similar studies conducted in rural [21] and urban Pakistan [26]. Whilst other studies have recorded higher prevalence rates [27, 28] these are potentially explained by contextual factors including hostile living conditions in refugee camps [28, 29] and limited health infrastructure in Pakistan’s tribal areas [27]. Compared with areas where the population was exposed to conflict or displacement the prevalence rate was relatively low [8, 30]. One explanation could be that in the preceding year life in the Swat valley has progressed from conflict to normalcy, and that the nature and severity of the military conflict was qualitatively less than what is faced by other populations. Another possible explanation could be that women in Swat might have displayed a degree of psychological resilience to conflict-related events, especially when the events occurred at a population level. Nevertheless, potentially traumatic events did have an independent effect on current levels of psychological distress.

When considering the prevalence of psychological distress one year after exposure to experienced PTEs a threshold effect was evident. This was explored through sensitivity analysis with PTEs as a dichotomous variable, which showed that the current psychological distress not manifesting until experiencing 3 or more PTEs. The impact of PTEs directly experienced was much greater than those either witnessed or heard. This indicates that while many women showed resilience to psychological distress, exposure to a higher number of directly experienced PTEs, even if occurred sometime ago, has a large impact on current mental wellbeing. Similar findings have been reported in studies in Southern Sudan where 8 or more traumatic events were independently associated with depression [8], and in Somali refugees living in the UK where cumulative pre-migration traumatic events were independently associated with anxiety and depression years later [31]. A meta-analysis of studies on refugees or conflict affected populations shows that cumulative exposure to PTEs was strongly associated with depression [30]. Other studies in conflict and post-conflict situations suggest similar association between major life events and mental health problems [32, 33].

Perceived family support, more likely in women living in extended families, was found to be a protective factor against psychological distress. This is supported by other studies in Pakistan which found nuclear family structure was an independent predictor of psychological distress during pregnancy [34], and extended family living a protective factor [22]. In another study on the general population in rural Pakistan, married women living in nuclear households were found to be more distressed as compared to those living in a joint family system [35]. This suggests the importance of family support, especially during pregnancy. This finding is important in light of the potential impact of humanitarian emergencies on social and family structures including family separation, loss of family members, disruption of social networks and traditional community resources [6] leading to significant disruption to sources of support.

## Conclusion

This is one of the first community based cross sectional survey in Swat valley, Pakistan to assess the prevalence of psychological distress during pregnancy in an area affected by conflict. Over a third of women show evidence of significant psychological distress. Exposure to potentially traumatic events remained independently associated with psychological distress 1 year after the conflict had ended, suggesting that conflict exposure may have long-term impacts upon maternal mental health. Similarly, psychological distress was also independently associated with three or more major life events in the year after the conflict. In addition, perceived family support and living in a joint family was found to be protective.

Given the impact of poor maternal mental health on the health outcomes of both the mother and child [2] it is important to focus on women of child-bearing age during and in the aftermaths of a conflict, as such exposure has long-term negative effects on maternal mental health, and potential negative impact on infant health and development. Improving family and social support by keeping families together may play an important role in protecting women’s psychological status during and following conflict. It would also be important, given the high rates of current psychological distress, to conduct further mixed-methods research exploring the complex associations and interactions between psychological distress and exposure to past and current stressors, as well as on culturally appropriate interventions that raise awareness and provide psychological support to these women.

## Additional files

Additional file 1: Table S1. PTEs and association with psychological distress. (DOCX 21 kb)
