# Supplementary material for: Psychological distress and its associations with past events in pregnant women affected by armed conflict in Swat, Pakistan: a cross sectional study
Source: Confl Health. 2015 Dec 10;9:37. doi: 10.1186/s13031-015-0063-4 (PMC4674905; doi:10.1186/s13031-015-0063-4)
Supplement: Additional file 1: Table S1. — PTEs and association with psychological distress. (DOCX 21 kb) [file 13031_2015_63_MOESM1_ESM.docx]

**Table S1: PTEs and association with psychological distress**

| **Potentially Traumatic events** | **Psychological distress**  **N= 133**  **N (%)** | **No psychological distress**  **N= 216**  **N (%)** | **P-value** |
| --- | --- | --- | --- |
| **Experienced PTEs** |  |  |  |
|  |  |  |  |
| Water and food scarcity | 81 (61) | 119 (55) | 0.287 |
| Inaccessible health care in need | 85 (64) | 121 (56) | 0.145 |
| Loss of shelter and logistics | 125 (94) | 189 (88) | 0.050 |
| Imprisonment | - | - | - |
| Dangerously wounded | - | - | - |
| War like situation | 110 (83) | 161 (75) | 0.075 |
| Brain washing | - | - | - |
| Sexual harassment | 3 (2) | 1 (1) | 0.157^1^ |
| Forcefully separation from others | 5 (4) | 4 (2) | 0.310^1^ |
| Being close to death | 76 (57) | 92 (43) | 0.008 |
| Forcefully separation from family | 6 (5) | 3 (1) | 0.090^1^ |
| Murder of family member or friend | 21 (16) | 23 (11) | 0.160 |
| Unnatural death of family member or friend | 27 (20) | 21 (10) | 0.005 |
| Stranger or strangers murder | 7 (5) | 3 (1) | 0.047^1^ |
| Kidnapping | - | - | - |
| Torture | 12 (9) | 5 (2) | 0.005 |
| **Witnessed PTEs** |  |  |  |
| Water and food scarcity | 25 (19) | 42 (19) | 0.881 |
| Inaccessible health care in need | 11 (8) | 27 (13) | 0.218 |
| Loss of shelter and logistics | 5 (4) | 10 (5) | 0.697 |
| Imprisonment | 36 (27) | 36 (17) | 0.020 |
| Dangerously wounded | 50 (38) | 76 (35) | 0.649 |
| War like situation | 1 (1) | 5 (2) | 0.414^1^ |
| Brain washing | 4 (3) | 9 (4) | 0.773^1^ |
| Sexual harassment | 2 (2) | 2 (1) | 0.637^1^ |
| Forcefully separation from others | 0 (0) | 1 (1) | 1.000^1^ |
| Being close to death | 2 (2) | 5 (2) | 0.713^1^ |
| Forcefully separation from family | 1 (1) | 1 (1) | 1.000^1^ |
| Murder of family member or friend | - | - | - |
| Unnatural death of family member or friend | 1 (1) | 4 (2) | 0.653^1^ |
| Stranger or strangers murder | 14 (11) | 19 (9) | 0.592 |
| Kidnapping | 7 (5) | 10 (5) | 0.789 |
| Torture | 1 (1) | 4 (2) | 0.653^1^ |
| **Heard PTEs** |  |  |  |
| Water and food scarcity | 18 (14) | 24 (11) | 0.499 |
| Inaccessible health care in need | 19 (14) | 29 (13) | 0.821 |
| Loss of shelter and logistics | 2 (2) | 7 (3) | 0.492^1^ |
| Imprisonment | 29 (22) | 33 (15) | 0.121 |
| Dangerously wounded | 31 (23) | 43 (20) | 0.450 |
| War like situation | 4 (3) | 13 (6) | 0.204 |
| Brain washing | 21 (16) | 25 (12) | 0.258 |
| Sexual harassment | 18 (14) | 14 (7) | 0.027 |
| Forcefully separation from others | 12 (9) | 12 (6) | 0.214 |
| Being close to death | 8 (6) | 10 (5) | 0.570 |
| Forcefully separation from family | 8 (6) | 9 (4) | 0.436 |
| Murder of family member or friend | 20 (15) | 20 (9) | 0.100 |
| Unnatural death of family member or friend | 6 (5) | 12 (6) | 0.668 |
| Stranger or strangers murder | 41 (31) | 58 (27) | 0.424 |
| Kidnapping | 19 (14) | 20 (9) | 0.148 |
| Torture | 22 (17) | 26 (12) | 0.235 |
